# Grounding implementation science in health equity for cancer prevention and control

**DOI:** 10.1186/s43058-022-00311-4

**Published:** 2022-06-03

**Authors:** Prajakta Adsul, David Chambers, Heather M. Brandt, Maria E. Fernandez, Shoba Ramanadhan, Essie Torres, Jennifer Leeman, Barbara Baquero, Linda Fleischer, Cam Escoffery, Karen Emmons, Montserrat Soler, April Oh, Ariella R. Korn, Stephanie Wheeler, Rachel C. Shelton

**Affiliations:** 1 Internal Medicine, School of Medicine, University of New Mexico, Albuquerque, NM USA; 2 Division of Cancer Control and Population Sciences, National Cancer Institute, Rockville, MD USA; 3 HPV Cancer Prevention Program, St. Jude Children’s Research Hospital, Memphis, TN USA; 4 Center for Health Promotion and Prevention Research, School of Public Health, University of Texas Health Science Center at Houston, Houston, USA; 5 Harvard T.H. Chan School of Public Health, Boston, MA USA; 6 East Carolina University, 2309 Carol Belk Bldg, Greenville, NC 27858 USA; 7 University of North Carolina, Chapel Hill, NC 27599 USA; 8 University of Washington, 3980 15th Ave. NE, Seattle, WA 98195 USA; 9 Fox Chase Cancer Center, Philadelphia, PA USA; 10 Rollins School of Public Health, Emory University, 1518 Clifton Rd, Atlanta, GA 30322 USA; 11 Harvard T.H. Chan School of Public Health, 677 Huntington Avenue, Boston, MA 02115 USA; 12 Ob/Gyn and Women’s Health Institute, Cleveland Clinic, Cleveland, OH USA; 13 Division of Cancer Control and Population Sciences, National Cancer Institute, National Institutes of Health, Bethesda, USA; 14 Cancer Prevention Fellowship Program, Implementation Science, Office of the Director, Division of Cancer Control and Population Sciences, National Cancer Institute, Bethesda, USA; 15 Gillings School of Global Public Health, University of North Carolina at Chapel Hill, 135 Dauer Drive, CB #7411, Chapel Hill, NC 27599 USA; 16 Department of Sociomedical Sciences, Columbia University, Mailman School of Public Health, 722 W 168th Street, New York, NY 10032 USA

**Keywords:** Health equity, Implementation science, Health disparities, Dissemination, Cancer prevention and control

## Abstract

**Background:**

The past decade of research has seen theoretical and methodological advances in both implementation science and health equity research, opening a window of opportunity for facilitating and accelerating cross-disciplinary exchanges across these fields that have largely operated in siloes. In 2019 and 2020, the National Cancer Institute’s Consortium for Cancer Implementation Science convened an action group focused on ‘health equity and context’ to identify opportunities to advance implementation science. In this paper, we present a narrative review and synthesis of the relevant literature at the intersection of health equity and implementation science, highlight identified opportunities (i.e., public goods) by the action group for advancing implementation science in cancer prevention and control, and integrate the two by providing key recommendations for future directions.

**Discussion:**

In the review and synthesis of the literature, we highlight recent advances in implementation science, relevant to promoting health equity (e.g., theories/models/frameworks, adaptations, implementation strategies, study designs, implementation determinants, and outcomes). We acknowledge the contributions from the broader field of health equity research and discuss opportunities for integration and synergy with implementation science, which include (1) articulating an explicit focus on health equity for conducting and reviewing implementation science; (2) promoting an explicit focus on health equity in the theories, models, and frameworks guiding implementation science; and (3) identifying methods for understanding and documenting influences on the context of implementation that incorporate a focus on equity.

**Summary:**

To advance the science of implementation with a focus on health equity, we reflect on the essential groundwork needed to promote bi-directional learning between the fields of implementation science and health equity research and recommend (1) building capacity among researchers and research institutions for health equity-focused and community-engaged implementation science; (2) incorporating health equity considerations across all key implementation focus areas (e.g., adaptations, implementation strategies, study design, determinants, and outcomes); and (3) continuing a focus on transdisciplinary opportunities in health equity research and implementation science. We believe that these recommendations can help advance implementation science by incorporating an explicit focus on health equity in the context of cancer prevention and control and beyond.

Contributions to the literature

- This paper presents a narrative review and synthesis of the conceptual and empirical literature at the intersection of implementation science and health equity research.
- We highlight opportunities identified at the health equity and context working group at the Consortium for Cancer Implementation Science to make a focus on health equity in IS foundational and explicit.
- Informed by the narrative review and identified opportunities by the action group, we offer recommendations that reflect the essential groundwork needed to promote bi-directional learning and synergy between the fields of implementation science and health equity research.

## Background

Over the past few decades, considerable public health and medical research has focused on identifying, understanding, and addressing health inequities, defined as unjust differences in health outcomes across population groups that are shaped by structural and social determinants of health [1–3]. The central role that these determinants play, in creating and reinforcing health inequities, has been particularly evident during the ongoing COVID-19 global pandemic [4, 5]. Consequently, there continue to be calls for a greater focus on health equity research that is social justice and action-oriented (e.g., focused on solutions), assets- or strengths-based, and actively seeks to promote and create conditions that facilitate the highest level of health for all [6, 7]. While there are recent conceptual, empirical, and methodological advances in Implementation Science (IS) [8], only a subset of this work has explicitly focused on promoting health equity [9], which we distinguish and operationalize using peer-reviewed literature in Table 1.

**Table 1:** Concepts, definitions, and considerations on what the literature says about health equity

Health differences	Health disparities / inequalities / inequities	Health equity / equality
• When differences are noted in health outcomes, between two groups based on a specific characteristic such as race, income, among other social or structural attributes of the population • Not all health differences warrant focused attention but health differences adversely affecting socially disadvantaged groups are particularly unacceptable because ill health can be an obstacle to overcoming social disadvantage [10]. • “Not all health differences are health disparities;” Paula Braveman suggests that health disparities are concerned with social justice (i.e., justice with respect to treatment of more advantaged vs. less advantaged socioeconomic groups in terms of healthcare) [1]	• Kawachi, I., and colleagues consider *health inequality* as a “generic term used to designate differences, variations, and disparities in the health achievements of individuals and groups.” [11] • Whitehead, M., suggests that “*inequalities*” in the European context have the same connotations of unfairness and injustice as the terms “*inequities*” [12] • *Health disparities* are differences that adversely affect socially disadvantaged groups; they are a specific subset of health differences that are relevant to social justice because they may arise from intentional or unintentional discrimination or marginalization and are likely to reinforce social disadvantage and vulnerability [7] • *Health disparity* is a more charged term; “to many a disparity implies an inequity or an injustice rather than a simple inequality” [13] • *Health disparities* emerge and persist through complex mechanisms that include socioeconomic, environmental, and system-level factors [14] • According to Kawachi, I. and colleagues, the term *health inequity* refers to those inequalities in health that are deemed to be unfair or stemming from some form on injustice [15] *How to determine whether a difference in health outcomes between groups is a disparity or not (in other words, are they unjust and unfair?)* • Which differences are inevitable, unavoidable, unnecessary, and unfair, will vary from country to country and from time to time [16]. • “Determining when a difference becomes a disparity may be problematic because a disparity is not measured directly, but rather as a residual or a difference between two groups, often only after other factors that might contribute to that difference have been statistically controlled for (more specifically in the context of racial and ethnic disparities” [13]	• “Equality is providing everyone with the same tools and resources. Equity is providing tools and resources based on needs that allow everyone the opportunity to be as healthy as possible.” [17] • “Health equity is the principle underlying a commitment to reduce, and ultimately, eliminate disparities in health and in its determinants, including social determinants.” [1] • “Health disparities and their determinants are the metric for assessing health equity, the principle underlying a commitment to reducing disparities in health and its determinants; health equity is social justice in health.” [7] • “Attainment of the highest level of health for all people. Achieving health equity requires valuing people equally with focused and ongoing societal efforts to address avoidable inequalities, historical and contemporary injustices, and the elimination of health and healthcare disparities” (Healthy People, 2020) • Equity in health care is the equal access to available care for equal need; equal utilization for equal need; equal quality of care for all [16]. • The crux of the distinction between equality and equity is that what we identify as a health inequity, depend on our own theories of justice, what we believe is society, and our reasoning on why we think health inequities exist [11].

Outside IS, there is a long and continuing history of scholarship related to health equity and the critical role of community-based participatory research (CBPR) approaches with racially and ethnically diverse communities [18–20]. This scholarship recognizes and establishes the role of social determinants of health, structural and interpersonal racism, historical trauma, and cultural-centeredness for implementing evidence-based interventions, programs, practices, and policies (referred to henceforth as EBIs) [21–23]. Despite the development of many EBIs and their demonstrated effectiveness among populations experiencing health inequities, widespread uptake of these interventions to improve population health outcomes has been limited [24–26]. There continues to be a limited focus on understanding the intersection of important social and structural dimensions that impact health and include age, disability, sexual orientation, gender identity, and geographic location (e.g., rural, urban) [18, 19], which can have implications for differing access to resources, opportunities, power, and/or obstacles that promote or hinder health. Furthermore, health equity and implementation researchers have largely operated in siloes, resulting in a lack of clarity and articulation of existing and potential synergies across these fields of research.

Recently however, there is a recognizable and explicit shift towards greater focus on health equity within IS [20–22]. For example, Baumann and Cabassa encourage integration of health equity across all IS aspects with a focus on reach from the start of the research projects, designing and selecting interventions with underserved populations, developing implementation strategies that help reduce disparities, advancing the science of adaptation, and using an equity lens for implementation outcomes [27]. Building on this, Brownson and colleagues propose establishing a scientific evidence base for health equity in IS, strengthening methods and measures, and focusing more on context—each requiring involvement of a wide range of stakeholders including researchers, program evaluators, funders, practitioners, communities, and advocates [9]. A recent article by Loper and colleagues operationalized equitable implementation as “including an explicit attention to the culture, history, values, assets and needs of the community and integration of these in to the principles, strategies, frameworks, and tools of IS” [23].

For this paper, consistent with prior definitions [24], we consider and refer to IS as a scientific field that incorporates perspectives from research, practice, and policy with the intent to bridge the gap between these and include a focus on both dissemination and implementation research. We argue that a greater focus is needed in IS on actively promoting health equity through explicit consideration of the social and structural injustices [25, 26]. Such an approach is critical and central to implementation science, with important implications for the types of EBIs we prioritize, disseminate, and implement in both our research and practice efforts. In this paper, we reflect on opportunities for cross-disciplinary, bi-directional, and collaborative learning between the fields of IS and health equity scholarship, and provide recommendations for enhancing a focus on health equity in the context of cancer prevention and control research.

### Consortium for Cancer Implementation Science (CCIS)

Research to promote health equity is an important area of focus for cancer prevention and control, which encompasses the “conduct of basic and applied research in the behavioral, social, and population sciences to create or enhance interventions that, independently or in combination with biomedical approaches, reduce cancer risk, incidence, morbidity and mortality, and improve quality of life” [28, 29]. To advance the mission of cancer prevention and control, the National Cancer Institute’s (NCI) Implementation Science team led the charge on creating the first Consortium for Cancer Implementation Science (CCIS) (previously called the Implementation Science Consortium in Cancer) as an innovative approach to produce “public goods” (considered to be papers, future meetings, workshops, and funding opportunities) and facilitate multidisciplinary collaborations in priority areas of research (i.e., technology, policy, learning health care systems, community participation, global health, health equity, complex/multilevel interventions, and study designs). Over two annual consortium meetings, in 2019 (in-person and virtual) and 2020 (only virtual), 522 researchers and practitioners from across the globe came together to engage in working groups dedicated to advancing the science of implementation [30, 31].

Members of the “Health Equity and Context” action group and their co-leads (authors, PA & RS for 2019 and PA, RS, AO, SW, AK for 2020) were charged with broadly reflecting on the intersection of IS and health equity research and exploring opportunities for synergy and learning across these fields, particularly in the context of cancer prevention and control. In preparation for the working group discussions (both in 2019 and 2020), co-leads conducted comprehensive scans of the literature that included a focus on IS and health equity research. This literature was synthesized and briefly presented to the action group for grounding, at the meetings in both 2019 and 2020. This paper is a result of the action group discussions that identified a need to review recent advances in IS and recognize the extensive history of health equity research that can inform IS through identified opportunities. Ninety unique participants (a mix of researchers and practitioners interested in IS and equity research, of varying levels of expertise) met over 2 days in 2019 and 2020 to learn, discuss priorities, and generate opportunities for incorporating a focus on health equity and context within IS. We present below the identified opportunities that are guided by a narrative review and synthesis of the extant literature, as ‘public goods’ to advance the science of implementation focused on cancer prevention and control.

### Opportunity 1: articulate an explicit focus on health equity for conducting and reviewing implementation science

Action group discussants identified a lack of explicit focus on health equity within IS and recognized the utility of developing a statement for the field about the importance of promoting and incorporating a focus on health equity. These discussions began in 2019 and were further emphasized in 2020, given the inequities highlighted with the ongoing global COVID-19 pandemic and national reckoning around structural racism [5]. In addition, discussants suggested addressing fundamental issues in the IS field that shape the nature of the research conducted, including provision of explicit language and definitions of health equity (attempted in Table 1) for funding announcements and review criteria in IS. Ultimately, the group sought to make equity a foundational grounding for the field which could ensure that we do not reinforce health inequities through our research [9, 32].

Discussants reviewed several opportunities with the goal of articulating an explicit focus on health equity in IS that could guide research and peer review. First, participants suggested developing a “checklist” that could guide considerations for health equity in implementation research. Examples of such equity-focused checklists, widely used in reporting studies (outside the field of IS) include the Preferred Reporting Items for Systematic Reviews and Meta-Analyses (PRIMSA)-Equity checklist [27, 33] and the Consolidated Standards of Reporting Trials (CONSORT)-Equity extension for improved reporting of health equity in randomized trials [34]. The Campbell and Cochrane Equity Methods group has lead the development of such checklists, in addition to the ongoing international effort to incorporate a focus on equity for the Strengthening the Reporting of Observational Studies in Epidemiology (STROBE) guidelines [35]. Similar to these checklists for study designs, participants recommended creating a IS-specific checklist to guide both researchers and funders as they proposed and reviewed studies focused on health equity.

Another opportunity identified to increase the focus on health equity in IS was the development of a values-orientation statement or a self-reflection guide that could be used by implementation researchers and practitioners to examine personal beliefs and biases related to health equity. Such an approach could also incorporate considerations for research institutions, community partners, and funding agencies, to operationalize how equity can be a fundamental consideration for IS studies [5, 9]. Such a guide could be informed by theories such as Critical Race Theory and the proposed Public Health Critical Race Praxis, which explicitly recognizes the societal and structural forms of racial discrimination and the consequences this may have for the effectiveness and sustainability of interventions [36, 37]. Finally, the group also recommended curating either exemplar research studies, case studies, or existing resources to reflect the integration of health equity in IS.

### Opportunity 2: promote an explicit focus on health equity in theories, models, and frameworks guiding implementation science

The action group discussions prioritized considering how equity could be integrated into existing implementation theories, models, and frameworks (TMFs). With approximately 150 TMFs [38, 39], implementation scientists have emphasized the value of TMFs and theorizing [39, 40] in research studies. Despite a wealth of TMFs, very few have included an explicit focus on improving health equity [41, 42] or been modified to incorporate a health equity focus [20]. Noting a recent trend, a few determinant frameworks have been revised critically to an explicit focus on health equity and include the Health Equity Implementation Framework by Woodward and colleagues [20], the race-conscious adaptation of the Consolidated Framework for Implementation Research [43], and application of an intersectionality lens to the Theoretical Domains Framework [44]. In terms of outcome frameworks, a recent extension of RE-AIM (Reach, Effectiveness, Adoption, Implementation, and Maintenance) model [41] now incorporates an explicit focus on equity and cost to facilitate the long-term sustainability and evolvability of EBIs over time [45]. Despite this progress, none of the TMFs comprehensively or explicitly incorporate or integrate established determinants of health equity (e.g., social determinants of health, structural discrimination, and racism) [5], which could be improved with continued efforts to engage health equity researchers.

The group also suggested conducting a scoping review of existing health equity frameworks that have a strong relevance for IS. This was recently highlighted in a scoping review of TMFs widely used in anthropology (e.g., theories of intersectionality, structural governance) and how they could inform a focus on health equity in ongoing and future implementation-focused studies [46]. Such integration of key health equity theories into IS can provide more depth in recognizing the role of structural and social determinants of health. Additionally, the group also recognized opportunities for bi-directional learning for health equity researchers to incorporate implementation considerations. For example, health equity research could benefit from the explicit considerations for implementation determinants at multiple socioecological levels and for partners involved in the delivery and implementation of EBIs [47], particularly as they relate to implementing change in organizational and policy contexts [48–50]. To integrate relevant work across these fields, the action group suggested convening a meeting of IS and health equity TMF developers and users to adapt existing frameworks to explicitly incorporate a health equity focus.

### Opportunity 3: identify methods for understanding and documenting influences on the context of implementation that incorporate a focus on equity

In planning for implementation, studies often assess and understand context [51, 52] that is operationalized across multiple levels of socioecological influence within healthcare settings [53]. Several discussants highlighted the importance of including an operationalization of health equity as part of “context” when studying the implementation of EBIs. Although the field needs to prioritize harmonizing the conceptualization of context across implementation studies, there is an urgent need to include a focus on historical and ongoing aspects of context that reflect structural roots of inequities such as structural racism [5] and historical trauma [54]. According to a recent review, only three TMFs in IS (i.e., Consolidated Framework for Implementation Research [51], Theoretical Domain Framework [55], and the integrated Promoting Action on Research Implementation in Health Services framework [56]) provide a specific definition of context [57], but without an explicit consideration for social and structural determinants of health. Even with considerable variations in the conceptualization of context, the most common dimensions identified were organizational support, financial resources, social relations and support, and leadership, suggesting that important gaps remain with respect to understanding and addressing health equity [57]. Outer context (i.e., external policies and incentives) which is an important but understudied aspect has an important influence on health equity, as it has important implications for distribution of resources and opportunities, and can set the broader environment in which organizational settings operate and provide services, with potential implications for inequitable implementation and delivery of care across resource-limited settings and underserved populations [58].

Understanding the impact of interventions on multiple socioecological levels is a priority for the NCI (https://healthcaredelivery.cancer.gov/mlti/) and refining methods for contextual assessment can be critical to supporting empirical studies that aim at promoting health equity. Contextual assessments can also inform the development and testing of implementation strategies across diverse settings and populations [59, 60]. Specifically, the group recognized the need to expand considerations for context beyond healthcare organizations to include interpersonal, community, and policy context. Recognizing ongoing efforts [61], the group proposed conducting a review of best practices for methods and measures that inform an assessment of contextual determinants and barriers/facilitators, particularly relevant to resource-limited settings and understudied populations. Such a review could inform the development of a methodology document for best practices that promotes an equity focus and includes specific measures that have been used for structural racism, discrimination, historical trauma, persistent poverty, and social deprivation, among others.

## Discussion

**Fig. 1:**
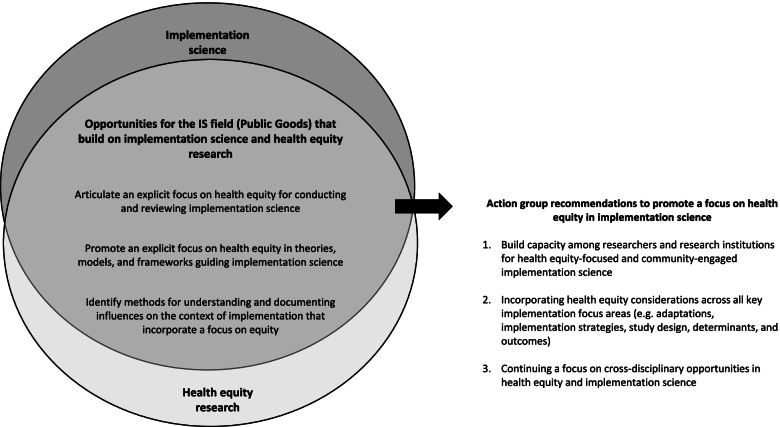
Opportunities and recommendations to promote health equity in implementation science, perspectives from the Health Equity and Context working group at the Consortium for Cancer Implementation Science

Taken together, the narrative review and the CCIS action group discussions highlight the growing importance of making health equity an explicit, central, foundational focus in IS. In advancing this work, our review makes it clear that there is a significant literature base around health equity [62–67]. This work can help IS researchers and practitioners reflect on and potentially evaluate whether or not their work reduces or inadvertently exacerbates inequities; additionally, for those without training and experience, there may be opportunities to partner with existing health equity experts, elevate and learn from their work, and ultimately advance research in this area. We acknowledge some limitations of this work. This was not a formal data collection effort for the participants in the working group; instead, discussions were undertaken as a participatory approach to engage researchers interested in health equity and context. In this vein, we may have missed some important perspectives. The intent of this review was to ground and contextualize the ideas and reflections during the working group discussions and we may have missed relevant literature in this narrative review. The authors of this paper, however, collectively support the opportunities identified by the CCIS Health Equity and Context action group related to focusing on health equity in conducting and peer-reviewing IS, in applying a health equity focus in IS-related TMFs, and when conducting contextual assessments. In order for these opportunities to advance the science of implementation, we present broad recommendations beyond the field of cancer prevention and control that can ensure progress in bi-directional learning and synergies between the fields of IS and health equity research (as shown in Fig. 1).

### Recommendation 1: build capacity among researchers and research institutions for health equity-focused and community-engaged IS

As a field, IS emphasizes the importance of multidisciplinary, stakeholder-engaged research for implementing EBIs, yet engagement with communities is not widespread in IS [8, 68–70]. CBPR is a broad term and reflects approaches, such as participatory action research, community-partnered, -engaged, and/or -based research, which exist along a continuum in terms of extent of community involvement, equitable resources, and decision-making power [71]. The central role of stakeholder and community engagement in promoting health equity has been the result of decades of work using CBPR approaches that prioritize community engagement in addressing inequities [63, 72]. In most instances, there is an emphasis on action, social justice, capacity building, and facilitating equitable partnerships [73]. As implementation scientists, partnering with community-engaged researchers can be critical in not rushing to “quick action”, and incorporating prior knowledge so as not to mischaracterize the inequities prevalent in the implementation context or the solutions to address them [74].

Researchers can incorporate CBPR approaches to make community and stakeholder voices integral to implementation research [68, 75]. While much of CBPR has focused on the community level, implementation scientists have the opportunity to expand this lens and apply it to a range of stakeholders at the policy, organizational, healthcare systems that include providers, healthcare administrators, and patients. The field of IS offers a natural fit for the commitment of participatory approaches which includes co-producing knowledge and action, as well as for systems change. An implementation brief put forth by the National Implementation Research Network proposes establishing a “co-creation environment” to promote collaborations among these various stakeholders and supports the capacity to promote the adoption, implementation, and sustainability of research evidence [76]. Such cross-disciplinary approaches have been used to build capacity and identify and implement breast cancer prevention-related EBIs across multiple jurisdictions in California, which led to the development of sustainable infrastructure models for primary prevention of breast cancer programs and implementation of research evidence [77, 78].

Recognizing the potential synergy between the fields of IS and CBPR, a special issue in the *Journal of Translational Behavioral Medicine* focused on “community-engaged dissemination or implementation research,” which included studies to implement EBIs within clinical or community-based settings using CBPR approaches [79]. From an equity perspective, it may be useful to take a “participatory implementation science” approach in IS while considering the levels of engagement between researchers and partners involved or impacted by the implementation efforts [68]. Consistent with capacity building, this approach ensures that diverse organizations and stakeholders have the knowledge, skills, and resources to implement and sustain EBIs [75] and is especially important as we strive to improve the quality and quantity of practice-based evidence to inform implementation efforts. As examples, frameworks such as Intervention Mapping [80, 81] and Transcreation Framework [82] incorporate co-creation of EBIs from the start in partnerships with communities, providing step-by-step processes to develop and evaluate interventions for the real-world community settings and account for important contextual factors and other influences in a population and setting. In fact, to effectively “design for dissemination” intervention researchers and/or program developers must assess, document, and address real-world needs, assets, and contextual realities of priority populations through consistent participatory engagement and co-learning [37, 83].

Pursuing health equity-oriented research can be demanding even for experienced researchers, and promoting training and opportunities to gain the skills and knowledge to do this work effectively is important for the advancing the field. Although there has been an increase (up to 55% in 2018) of implementation scientists engaging with stakeholders, there continues to be a lack of specificity around the methods of engagement and a misalignment with academic priorities of tenure and promotion [84, 85]. Determining the required competencies for researchers focused on health equity may serve as important next steps [86], with a specific focus on IS [87]; further, evaluating perceptions of researchers working at the intersection of health equity and IS might allow for a comprehensive understanding of the motivations and barriers to proposing and conducting equity-oriented IS. Efforts at the national level [88] are needed to provide career development opportunities that prioritize recruitment and retention of scholars from under-represented groups and support scholars conducting or interested in learning how to conduct equity-oriented research.

### Recommendation 2: incorporate health equity considerations across key implementation focus areas (e.g., adaptation, implementation strategies, study design, determinants, and outcomes)

With growing number of EBIs, existing guidance has helped researchers identify where along the translational pipeline to intervene, when addressing health inequities. For example, Chinman et al. outlined a decision tree to help researchers select an effectiveness and/or implementation trial design, depending on the goal of either identifying, understanding, or addressing disparities/inequities [89]. McNulty et al. highlight methodological approaches for conducting implementation research to advance health equity that includes a paradigm of focusing on existing data with systems science methods, including populations with inequities to design new studies, or a focus exclusively on populations experiencing inequities [90]. Incorporating a focus on health equity has important implications for the research questions being answered and whether or not the outcomes of an implementation effort would differ based on the incorporation of diverse settings and populations. Despite the focus on implementation, most interventions are not optimized prior to delivery and may influence both the effectiveness and the implementation of the EBIs. Consequently, a significant focus in the field of IS has been on studying the adaptation of interventions as a potential opportunity to enhance effectiveness, improve fit in the implementation context, and increase the likelihood of sustainability [91].

The science of adaptation has tremendous promise in promoting health equity [21, 67]. Although adaptations can improve the EBI’s effectiveness [92–94], it could also render the intervention less effective if key components are removed [95, 96]. For these reasons, it is important to specifically and systematically report considerations for why and how the adaptations were made, especially when working with resource-limited settings and underserved populations. Frameworks such as the Framework for Reporting Adaptations and Modifications – Expanded (FRAME) [97] support the systematic documentation of adaptation and can contribute to understanding the impact of adaptation on health equity. Complementary to this effort, a scoping review identified 13 frameworks that were consolidated to identify eight commonly used adaptation steps or processes to guide researchers and practitioners in the field [98]. A synthesis of the key steps required for facilitating planned adaptations provides an important contribution to the field; however, evidence is still needed to determine whether these steps need to be modified based on the type of intervention or setting. It is also critical to ensure that the guidance offered is actionable in organizations that may not have staff with formal training in public health, health disparities, or IS, which also presents a unique opportunity to test these adaptation frameworks with respect to health equity goals.

Since the push in 2013 for reporting and specifying implementation strategies [99], the field has progressed tremendously with the introduction of the Expert Recommendations for Implementing Change (ERIC) taxonomy [100], with calls for more focus on effectiveness research evaluating discrete implementation strategies [59] as well as mechanisms through which strategies influence implementation and health outcomes [60]. What is often missing in this discussion, however, are implications on advancing equity [46]. There is an urgent need to explicitly evaluate whether certain implementation strategies are effective at promoting equity or reducing inequities [9, 59]. Questions to consider regarding strategies may be as follows: (1) which are more acceptable and feasible in resource-limited settings or underserved populations; (2) which are more appropriate or effective in promoting equity; and (3) what are the specific mechanisms through which they operate to promote equity when creating change? Implementation Mapping as a methodology provides a systematic process for planning (or selecting and tailoring) implementation strategies and explicitly integrates both a participatory and an equity perspective [101]. Although future work is needed to validate this method, the process guides the engagement of the community throughout the process and considers the possibility of tailoring (differentiating) specific strategies depending on the unique needs of subgroups within the populations of interest. Future research is needed to integrate an explicit attention to the culture, context, history, and needs of the communities, when prioritizing the selection and tailoring of strategies that can facilitate the effective implementation and sustainment of EBIs among historically and systematically underserved populations, supported in the projects designed at optimizing strategies [102], in the recently funded Implementation Science Centers in Cancer Control [103].

### Recommendation 3: continuing a focus on cross-disciplinary opportunities in health equity and implementation science

True to the spirit of IS, we believe in continuing conversations with researchers and practitioners across disciplines, to generate cross-disciplinary, multi-sector solutions that promote health equity. The recent calls from the National Institutes of Health, and the NCI in particular, provide important opportunities to provide information on innovative and provoking ideas to promote health equity and address inequities and promote a diverse biomedical workforce [104, 105]. Emerging cross-disciplinary conversations among implementation, policy scientists, and economists showcase the added value of and the promising strategies to promote equity while advancing the science of implementation [49]. Researchers will also need to seek out opportunities to engage communities across a range of social and structural dimensions (i.e., race/ethnicity, socioeconomic status, age, disability, sexual orientation, gender identity, and geographic location (e.g., rural, urban)). In 2020, the CCIS steering committee promoted the inclusion of practitioners and policymakers as essential partners for integrating IS and health equity research to produce meaningful and sustained impact [30]. Such integration is aligned with Health in All Policies initiatives [106, 107], multisectoral approaches [108, 109], and promotion of community engagement in IS [70], all of which are crucial in generating relevant, feasible, and sustainable solutions to promoting health equity.

While multidisciplinary collaborations will be critical, it is also important to recognize the growing research around implementing interventions across diverse populations (i.e., among indigenous Maori community in New Zealand [110]), settings (i.e., applications of CFIR in low-and middle-income countries [111]), research institutions (i.e., among Clinical and Translational Science awardees [22] and offices of community outreach and engagement in NCI-designated cancer centers [112]), and for non-cancer-focused research priorities (i.e., cardiovascular disease [113, 114], genomic medicine [115]). Lessons learned from such applications of IS methods, TMFs, and study designs can highlight innovative and effective strategies to promote health equity. Even when new EBIs are developed and tested for diverse populations and settings, alignment with or consideration of IS will be critical. Guidance from the recent theoretical advances, such as the ConNECT framework, put forth collectively by the Society for Behavioral Medicine’s Ethnic Minority and Multicultural Health Special Interest group, can be key in integrating context, fostering a norm of inclusion, ensuring equitable diffusion of innovations, harnessing communication technology, and prioritizing specialized training [116]. Research networks like CPCRN that are building capacity through trainings on “Putting Public Health Evidence into Action” can serve as additional opportunities for diverse stakeholders to understand intervention impact and utility across the implementation continuum [117, 118].

Inventive and resourceful collaborations are essential in tackling evolving challenges on addressing the adverse influences of multiple socioecological levels on health and healthcare [119], study the impact of complex interventions [120], and achieve sustainability for potential impact and value/return on investment in interventions [121]. Decades of intervention research produced in controlled environments, coupled with traditional linear approaches, have failed to achieve the intended population health outcomes in the real-world settings [53, 122]. Although the complexity associated with interventions needed to change health outcomes has a long history of recognition, only recently have there been efforts in improving the capacity in terms of skills and collaborations [80, 123]. More often than not, research takes place in settings that have sufficient resources enabling them to participate in research without disrupting the provision of clinical, community, or public health services. When funding ends, implementing and sustaining interventions often requires additional resources in terms of staff, time, and money, or scaling out of the intervention to reach diverse settings [124]. The current funding climate requires researchers to think creatively and keep sustainability at the center of all decisions related to research and partnerships its potential impact and potential value/return on investment, in terms of practice [91]. Implementation researchers could also benefit and contribute towards health equity goals by proposing studies using pragmatic designs [125], incorporating multilevel analyses [53], and engaging in the science of complex interventions [126].

## Conclusions

The ongoing COVID-19 pandemic has made apparent the longstanding structural inequities and systems that create widespread health inequities at a population level [127]. Addressing these will require a sharp focus on understanding the varied mechanisms by which underlying fundamental injustices (e.g., racism, discrimination) and social determinants of health influence, and in some case hinder, the implementation of policies and programs for populations. The complex, multilevel array of factors that contribute to health inequities also have important implications for the implementation of EBIs across diverse settings/populations [128], though they have not always been explicitly identified as such [129, 130]. While we recognize the complexity in addressing these structural, upstream challenges, the ongoing public-health crises present important opportunities for changing systems on a broad scale by taking a proactive approach to incorporate a focus on health equity in ongoing and future implementation studies [25, 131].

Myriad factors within the field of IS contribute to a lack of explicit focus on health equity [84]. However, the underlying premise of the IS field is to make sure EBIs have widespread impact and that they benefit populations representing diversity with respect to race/ethnicity, socioeconomic status, age, disability, sexual orientation, gender identity, and geographic location (e.g., rural, urban) [132, 133]. Identifying and integrating research that reflects the long history of equity research can propel IS to be more inclusive of and committed to health equity. On the other hand, a continued lack of explicit focus on equity in the field can make it difficult to track whether health equity goals are achieved and increase the risk of expanding the translation gap with greater exclusion of those who may benefit most from interventions to improve health. Increasing health equity is within reach, if we commit to building capacity and continue to collaborate with multidisciplinary researchers, practitioners both in health and non-health sectors, policy makers, and most importantly individuals with lived experiences, with a reframe of our theories, frameworks, and methods with equity at the forefront. Beginning with the acknowledgement that health is not distributed across all populations equally, we can work collectively to promote a science for implementation that benefits all, with no one left behind.

## Data Availability

All meeting reports from the 2019 and 2020 Consortium for Cancer Implementation Science (previously known as the Implementation Science Consortium in Cancer) are available at: https://cancercontrol.cancer.gov/is/initiatives/iscc

## Abbreviations

IS: Implementation science
NIH: National Institutes of Health
ISCC: Implementation Science Consortium in Cancer
CCIS: Consortium for Cancer Implementation Science
NCI: National Cancer Institute
EBI: Evidence-based intervention
TMF: Theories, Models, and Frameworks
